# Cough remedy leading to chronic cough: An unusual airway foreign body‐Clove

**DOI:** 10.1002/rcr2.1130

**Published:** 2023-03-23

**Authors:** M. D. Varunn, R. Jayakumar, Chandrakant Tarke, Kishan Srikanth Juvva, D. Senthil

**Affiliations:** ^1^ Department of Respiratory Medicine PSG Institute of Medical Sciences and Research, Peelamedu Coimbatore India; ^2^ Department of Pulmonology Apollo Hospitals Hyderabad India; ^3^ Department of Pulmonology KIMS Hospital Hyderabad India

**Keywords:** airway foreign body, bronchoscopy, clove, cough remedy

## Abstract

The spectrum of presentation of airway foreign body can vary from having mild symptoms to sudden death. Smaller foreign body in distal airways, especially if the patient is unaware of aspiration can result in chronic symptoms mimicking asthma. Clove, has been used traditionally for its medicinal values and commonly used as a cough remedy. In this case series, we report four cases of this unusual airway foreign body which were essentially consumed with an intention to prevent cough, but unfortunately became the reason for their cough.

## Graphical abstract

Clove has been used traditionally for its medicinal values and is commonly used as a cough remedy. In this case series, we report 4 cases of this unusual airway foreign body which were essentially consumed with an intention to prevent cough, but unfortunately became the reason for their cough.

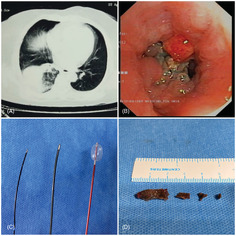

## INTRODUCTION

Airway foreign body can be life‐threatening, if appropriate care is not given early. It is one of the leading causes of unintentional death, especially in children under the age of 16.^1^ Complete obstruction of major airways can cause asphyxia resulting in sudden death. However smaller foreign bodies that reach the distal airways can be asymptomatic or results in cough, breathlessness and wheezing. The acuteness of the problem depends on multiple factors—age, foreign body type, whether the patient was aware of aspiration, location of the aspirated material, degree of obstruction and access to Interventional Pulmonology unit.^2^ Based on the physical properties, foreign bodies can be classified into three types—organic (e.g., seeds, vegetables, and nuts), inorganic (e.g., plastics and pills) and metallic (e.g., coins, pins, and implants).

In this case series, we report four cases of unusual airway foreign body which were essentially consumed with an intention to prevent cough, but unfortunately became the reason for their cough.

## CASE 1

**FIGURE 1:**
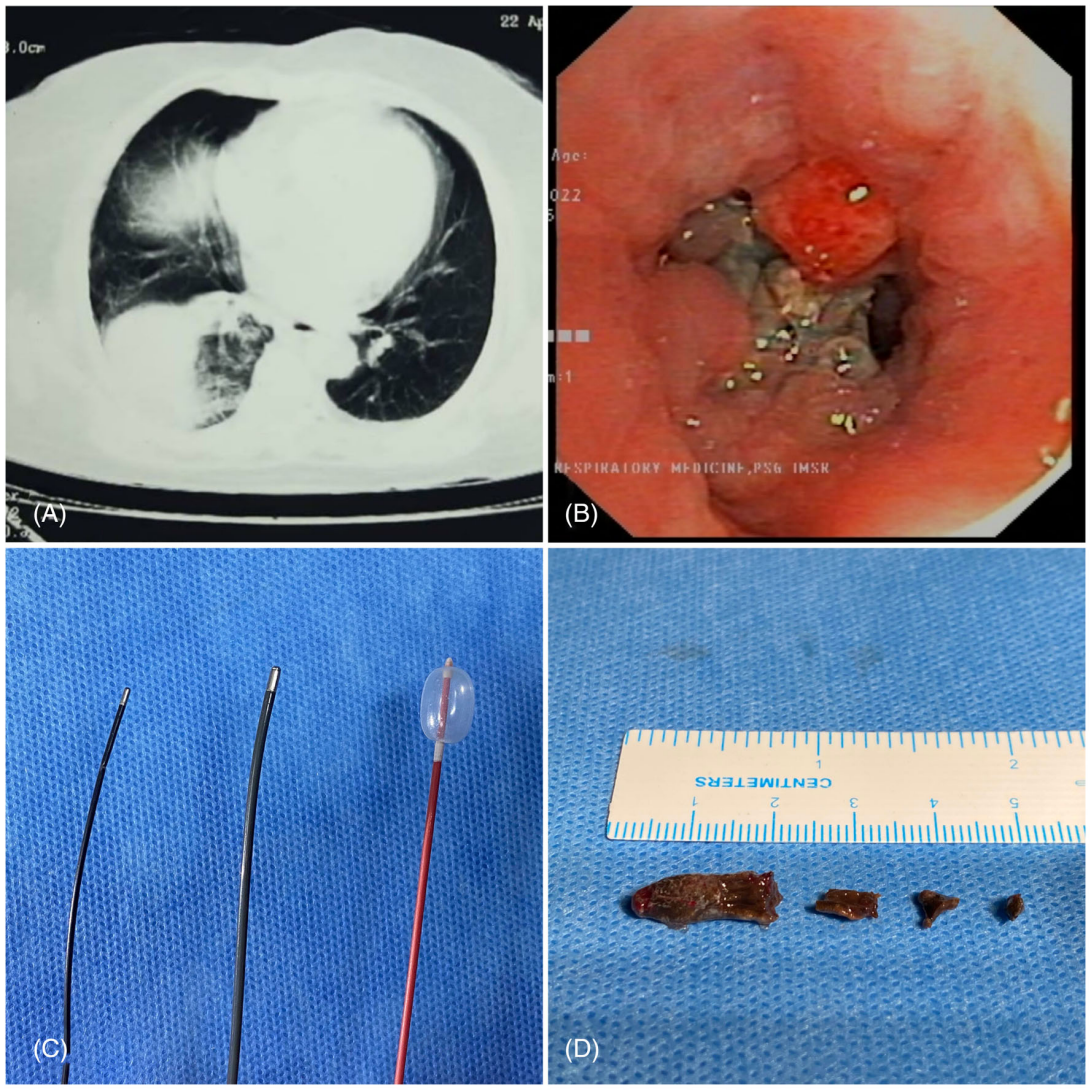
(A) CT chest showing mass like consolidation in the right lower lobe, (B) endobronchial view showing impacted foreign body material with granulation tissue in RB10, (C) 1.1 mm, 1.7 mm Erbe cryoprobe and 4 Fr Fogarty balloon, and (D) retrieved clove

A 53‐year‐old female presented with complaints of cough for 1 month. Chest X‐ray showed a right lower zone homogenous opacity. She was evaluated elsewhere with a CT chest which showed a right lower lobe mass‐like consolidation (Figure 1). A diagnostic bronchoscopy done initially showed a suspected endobronchial lesion in the right lower lobe bronchus, forceps biopsy of which was inconclusive. The patient was referred to PSG Institute of Medical Sciences and Research, Coimbatore for further management. A diagnostic bronchoscopy (Olympus BF 1 T180/OD 6.0 mm/ID 3.0 mm) was done which showed granulation tissue in the RB10 segment of the right lower lobe. As there was bleeding from the lesion during the initial diagnostic bronchoscopy, the patient was intubated using a rigid bronchoscope (Effer Dumon 12 mm Tracheal barrel) under general anaesthesia for a better airway access. The flexible video bronchoscope was used with rigid bronchoscopy as a conduit. The tissue was devitalised using 1.7 mm cryoprobe (Erbe). A blackish material was noted distally. Initial attempts to remove with 1.1 mm cryo (Erbe) failed, which was later removed by distal balloon dilatation technique using a 4 Fr fogarty balloon. The extracted foreign body was identified to be a clove. Post‐procedure, the patient admitted that she developed the habit of chewing clove daily during the COVID pandemic (almost for 1 year when she presented with symptoms) for its beneficial effects in preventing cough.

## CASE 2

**FIGURE 2:**
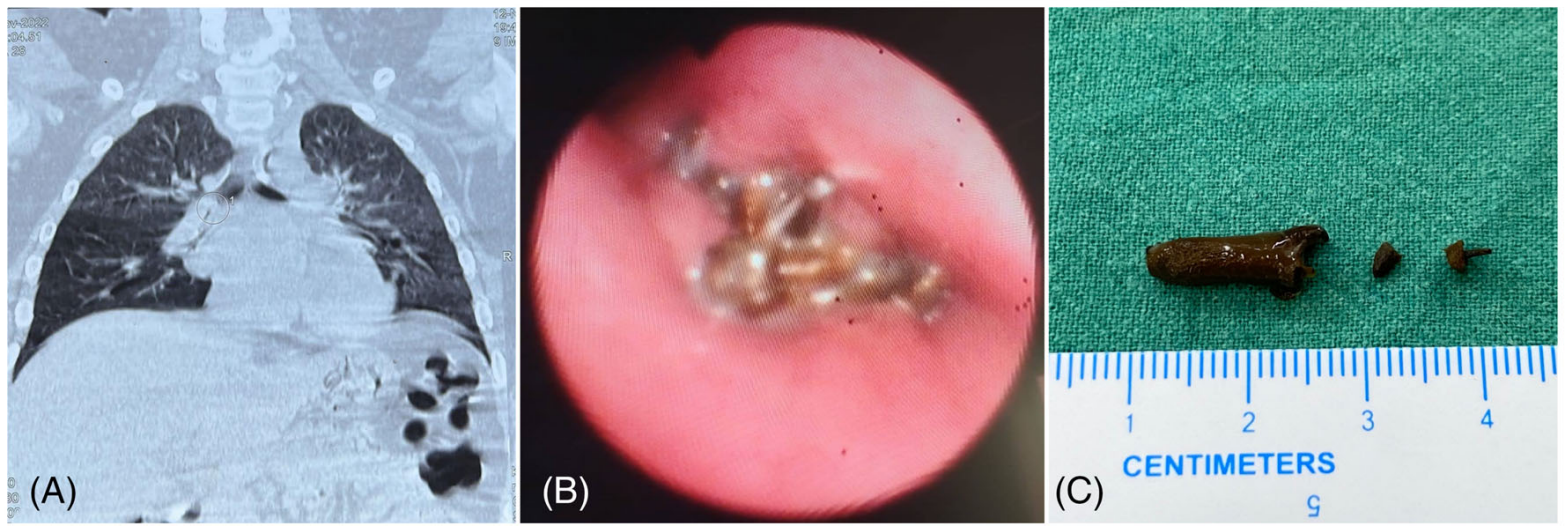
(A) Coronal plane CT showing obstruction in the right intermediate bronchus, (B) endobronchial view at the level of right main bronchus showing impacted clove with granulation tissue in the proximal right intermediate bronchus, and (C) retrieved clove

A 9‐year‐old boy, who is an asthmatic since childhood presented with recurrent cough for the past 6 months. He was regular in taking inhaled corticosteroids, however, he had recurrent exacerbations. Chest X‐rays done initially were within normal limits. A CT scan done for evaluation of recurrent exacerbations showed an endobronchial foreign body material in the right intermediate bronchus, for which the patient was referred to PSG Institute of Medical Sciences and Research, Coimbatore. A flexible fibre optic bronchoscopy (Olympus BF PE2/OD 4.9 mm/ID 2.2 mm) was done under general anaesthesia (via Endotracheal tube) showed a brownish material which was occluding the right intermediate bronchus with surrounding granulation tissue (Figure 2). The granulation tissue was devitalised using 1.1 mm Erbe cryoprobe and then the material was removed using the same. It was found to be a clove that the boy has consumed as a home‐remedy for his recurrent cough.

## CASE 3

**FIGURE 3:**
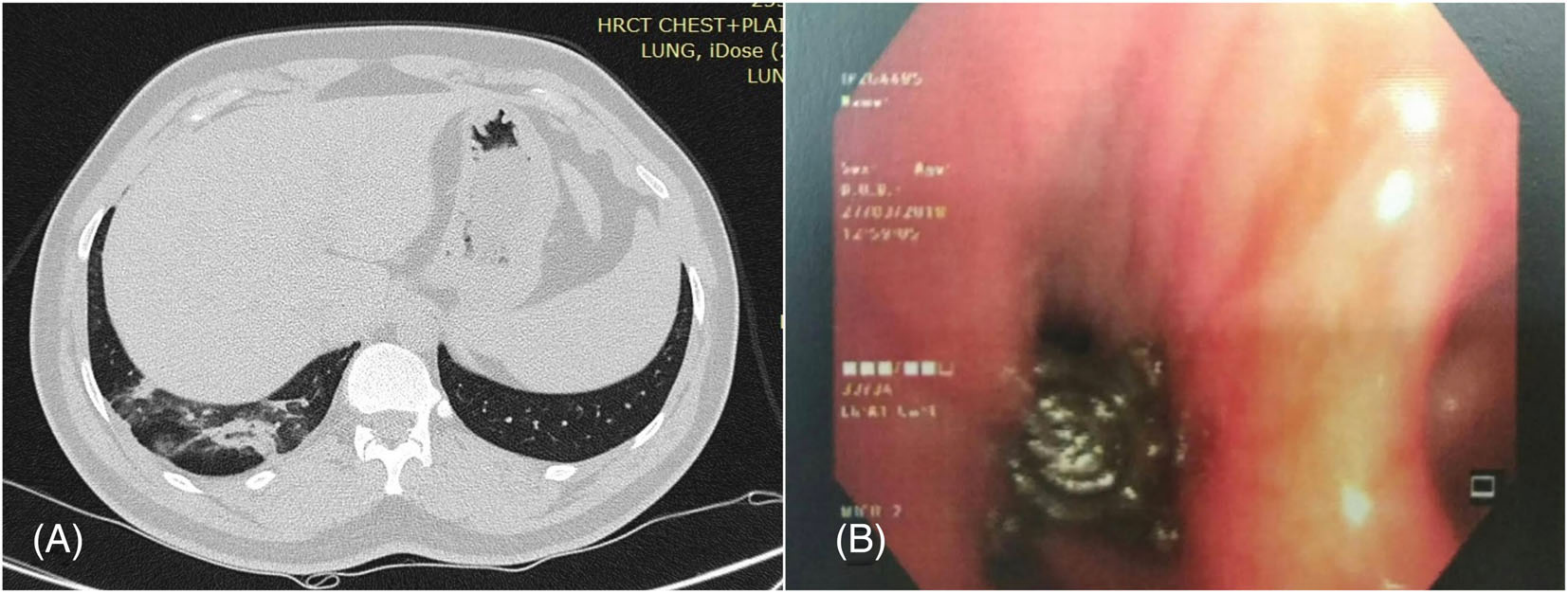
(A) Axial CT showing right lower lobe patchy consolidation and (B) foreign body in the right lower lobe medial basal segment

A 37‐year‐old male presented to Apollo Hospital, Hyderabad, with a history of cough for 1 month. On evaluation, chest X‐ray showed right lower zone haziness, CT showed right lower lobe segmental collapse with consolidation (Figure 3). On Bronchoscopy (Olympus BF 1TH190/OD 6.2 mm/ID 2.8 mm) with local anaesthesia, the right lower lobe medial basal segment showed a foreign body with purulent secretions. The foreign body was removed using reusable biopsy forceps (2.6 mm) which was found to be clove. The patient had the habit of eating clove for its health benefits, but there was no obvious history of aspiration.

## CASE 4

**FIGURE 4:**
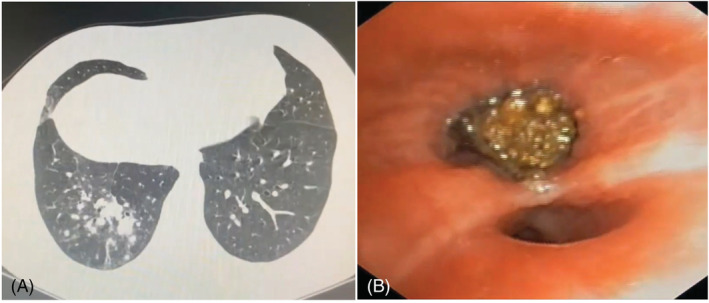
(A) Axial CT showing right lower lobe patchy consolidation and (B) foreign body in the right lower lobe lateral basal segment

A 50‐year‐old male with no comorbidities came to KIMS hospital, Hyderabad, with complaints of cough and fever for 10 days. He was treated with antibiotics for 1 week despite which he had persistent symptoms. Chest X‐ray showed right lower lobe infiltrates. CT chest showed patchy consolidation in the posterior and lateral basal segments of right lower lobe (Figure 4). Under local anaesthesia, a flexible video bronchoscopy (Olympus BF 1 T150/OD 6.0 mm/ID 2.8 mm) was done which showed a brownish material in the lateral basal segment. It was removed using reusable alligator forceps (2.6 mm) and it was a clove. The patient had a habit of chewing a clove before he goes to bed for the last 10 years.

## DISCUSSION

The type of airway foreign body varies based on the culture, religion, and food habits of the patient.^2^ Organic materials like peanuts were the most common foreign body in the western population, while bones were reported in south East Asian reports.^3^ In muslim females who wear hijabs, aspiration of scarf pins held in mouth while wearing hijab is commonly reported.^4^ As children are curious about the surroundings, they might place inappropriate objects like toys, plastic objects, coins which can get aspirated.

Clove (*Syzygium aromaticum*) is an aromatic flower bud of the tree which belongs to Myrtaceae family, has origin from Indonesia.^5^ It is a spice that is primarily used for its aroma as a flavouring agent and certain consumer products like cosmetics and toothpaste. Eugenol, which is a major component of clove imparts its taste. The US Food and Drug Administration has confirmed that the use of clove as a food supplement is safe and, traditionally it has been in use for centuries for its potential medical benefits. The anti‐inflammatory and analgesic property of eugenol is effective for toothache, sore throat, cough and certain of pain.^6^, ^7^ They also have potential risks like liver damage, seizures and clotting disorders if consumed in large quantities.

The characteristic features of clove make it a peculiar airway foreign body. It can get wedged at the level of segmental airways because of its nail‐like shape. It absorbs water and swells up, eventually making it hard to remove. It also gets friable and breaks easily while removing. Moreover it stimulates intense inflammation, resulting in granulation tissue which makes it more challenging. In the four cases described above, the first two cases had probable history of aspiration 2 months ago resulting in formation of granulation tissue, where we had to use cryo for devitalisation. To avoid disintegration, a fogarty balloon can be inflated distally and gently pulled, which can then be removed using forceps, cryo probe or foreign body basket. This ‘distal balloon dilatation’ technique will be useful in any impacted airway foreign body.

There are only a few anecdotal reports describing this rare airway foreign body. This is the first case series in the literature describing clove as an airway foreign body. To the dismay, all the four patients had the habit of chewing clove for its medicinal benefits in preventing or suppressing cough, but became the reason for their cough.

Clove's health benefits are undeniable, however, the method by which it is consumed is important. Some of them chew it before bedtime to prevent nocturnal cough. Due to its analgesic properties, chewing and retaining it in mouth can increase the risk of aspiration, especially when there is cough. Awareness on how to consume is important to prevent the potential complication of it becoming a rare airway foreign body.

## AUTHOR CONTRIBUTIONS

All persons listed as authors were involved in patient care.

## CONFLICT OF INTEREST STATEMENT

None declared.

## ETHICS STATEMENT

The authors declare that appropriate written informed consent was obtained for the publication of this manuscript and accompanying images.

## Data Availability

Data sharing not applicable to this article as no datasets were generated or analysed during the current study.
